# Molecular Imaging to Predict Response to Targeted Therapies in Renal Cell Carcinoma

**DOI:** 10.1155/2017/7498538

**Published:** 2017-04-09

**Authors:** Ingrid Leguerney, Ludovic de Rochefort, Marie Poirier-Quinot, Alexandre Ingels, Xavier Violas, Sandra Robin, Paule Opolon, Rose-Marie Dubuisson, Stéphanie Pitre-Champagnat, Philippe Robert, Nathalie Lassau

**Affiliations:** ^1^IR4M, UMR 8081, Paris-Sud University, CNRS, Bâtiment 220, rue Ampère, 91405 Orsay Cedex, France; ^2^Gustave Roussy, 114 rue Edouard Vaillant, 94805 Villejuif Cedex, France; ^3^Service Urologie, Hôpital Bicêtre, 78 rue du Général Leclerc, 94270 Le Kremlin-Bicêtre, France; ^4^Experimental Imaging, MRI Unit, Research Division, Guerbet, 93600 Aulnay-sous-Bois, France

## Abstract

Molecular magnetic resonance imaging targeted to an endothelial integrin involved in neoangiogenesis was compared to DCE-US and immunochemistry to assess the early response of three different therapeutic agents in renal cell carcinoma. Human A498 renal cells carcinoma was subcutaneously inoculated into 24 nude mice. Mice received either phosphate-buffered saline solution, sunitinib, everolimus, or bevacizumab during 4 days. DCE-US and molecular MRI targeting *α*v*β*3 were performed at baseline and 4 days after treatment initiation. PI, AUC, relaxation rate variations Δ*R*2^⁎^, and percentage of vessels area quantified on CD31-stained microvessels were compared. Significant decreases were observed for PI and AUC parameters measured by DCE-US for bevacizumab group as early as 4 days, whereas molecular *α*v*β*3-targeted MRI was able to detect significant changes in both bevacizumab and everolimus groups. Percentage of CD31-stained microvessels was significantly correlated with DCE-US parameters, PI (*R* = 0.87, *p* = 0.0003) and AUC (*R* = 0.81, *p* = 0.0013). The percentage of vessel tissue area was significantly reduced (*p* < 0.01) in both sunitinib and bevacizumab groups. We report an early detection of neoangiogenesis modification after induction of targeted therapies, using DCE-US or *α*v*β*3-targeted MRI. We consider these outcomes should encourage clinical trial developments to further evaluate the potential of this molecular MRI technique.

## 1. Introduction

Renal cell carcinoma (RCC) is a chemotherapy and radiation resistant cancer, and its management had been limited to surgical extirpation for a long time. The development of targeted therapies during the last decade has led to a tremendous improvement of patient's survival in the setting of metastatic spread. The first randomized control trial leading to food and drug administration approval to use targeted therapy in renal cell carcinoma was published in 2007 [1] when sunitinib appeared superior to interferon-*α* regarding progression-free survival and response rates. Since that date, many new agents have been approved targeting either vascular endothelium growth factor receptor (VEGFR), sunitinib, sorafenib, pazopanib, bevacizumab, and axitinib, or the mammalian target of rapamycin (mTOR) pathways: temsirolimus and everolimus. With this new drug arsenal, patients can receive several lines of treatment during their follow-up. For a better disease control, it is paramount to evaluate as early as possible the tumor response to one agent in order to decide if this treatment can be prolonged or switched to another one. Monitoring therapeutic response using preclinical tumor models allows improving our knowledge of the therapies benefits depending of a wide range of tumor models. DCE-US has been used for many years for the evaluation of therapies [2–5]. Over recent years, molecular imaging has become established in preclinical research for the detection of inflammation and angiogenesis allowing a broad field of applications [6–9]. This emerging technique allows imaging more specifically some cancers by attaching molecules targeting pathways involved in cancer development to a contrast agent [10]. This association between imaging signal and molecular expression enables a better and faster monitoring of drug response.

Several preclinical studies have reported the potential interest of molecular magnetic resonance imaging targeting the *ανβ*3 marker to analyze neoangiogenesis in melanoma, hepatocellular carcinoma, or peripheral vascular diseases [11–13]. The aim of this study was to propose a multimodal imaging protocol comprising dynamic contrast enhanced ultrasonography (DCE-US) and magnetic resonance imaging (MRI) that provide complementary information on the status of a tumor. The study purpose was to compare imaging methods to predict the early response to targeted agents validated in renal cell carcinoma. We compared the response of three different agents: sunitinib, bevacizumab, and everolimus using DCE-US, molecular MRI targeting *α*v*β*3, an endothelial integrin involved in neoangiogenesis, and immunochemistry.

## 2. Materials and Methods

All experiments were conducted in agreement with the European Convention for the Protection of Vertebrate Animals used for experimental and other scientific purposes and were approved by the Animal Research Committees, CEEA-26 and CEEA-44 (registered by the French research ministry), where the experiments take place.

Two authors (Philippe Robert and Xavier Violas), employees of Guerbet (Aulnay-sous-Bois, France), provided the P04000 contrast agent for the MRI experiments (patent application US 2015/0320889 A1 untitled “vectorised magnetic emulsion” filed on Nov. 12, 2015). The other authors, who are not employees or consultants for Guerbet, retained full control of the data and information submitted for publication.

### 2.1. Mice and Tumor Model

Twenty-four female immunodeficient nude mice (6–8 weeks old) were bred and housed in the Animal Care Facility at Gustave Roussy (Villejuif, France), in accordance with institutional guidelines for animal welfare. Human A498 renal cells carcinoma (ATCC-HTB-44, American Type Culture Collection, Manassas, USA) was cultured in EMEM (Eagle's Minimum Essential Medium, Gibco Life Technologies, Gaithersburg, MD, USA) supplemented with 10% FBS (Fetal Bovine Serum). The experiments started 30 days after 3 × 10^6^ cells (cell passage 4) in 100 *μ*L Corning® Matrigel® Growth Factor Reduced (GFR) Basement Membrane Matrix (Corning, Tewksbury, USA) were subcutaneously inoculated into the right flank of the mice.

### 2.2. Drug Therapy

The mice were randomized into four groups of six mice each. The control group received a daily administration of phosphate-buffered saline solution. The treated groups received either sunitinib (SUTENT, Pfizer, New York City, New York, USA) at a dose of 40 mg/kg, everolimus (AFINITOR, Novartis, Basel, Switzerland) at a dose of 10 mg/kg, or bevacizumab (AVASTIN, Genentech/Roche, Basel, Switzerland) at a dose of 5 mg/kg. All the compounds were administered daily during 4 days by oral gavage using soft cannulas, except for the bevacizumab injected intraperitoneally for 2 consecutive days.

### 2.3. Imaging Sessions

Evaluation of treatment efficacy was evaluated by two imaging techniques, dynamic contrast enhanced ultrasonography (DCE-US) and molecular MRI at both baseline (day 0, D0) and 4 days after treatment initiation (day 4, D4). For each imaging session, animals were anesthetized by inhalation of isoflurane (2% in air at 1.5 L/min) and body temperature was maintained constant during the acquisitions. Acquisition parameters were optimized for US and MRI and remained the same during the whole study.

**Figure 1:**
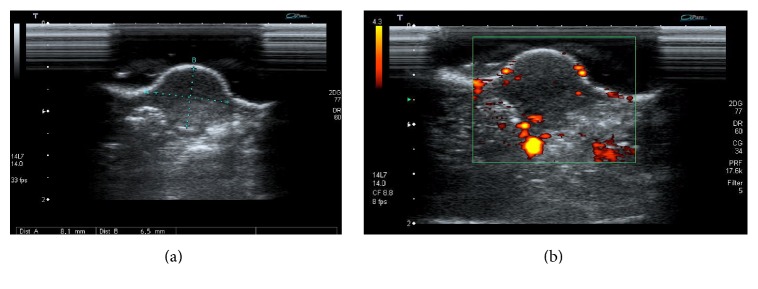
(a) Tumor volume evaluated in B-mode by ultrasonography. (b) Example of Doppler imaging for the assessment of the number of intratumor vessels.

The tumors were first imaged by 2D-ultrasonography using an Aplio scanner (Toshiba, France) to measure the tumor volume by B-mode imaging (Figure 1(a)) and to evaluate the number of intratumor vessels using power Doppler mode (Figure 1(b)) with a 14 MHz probe (PLT-1204AT, Toshiba, France). The complete imaging technique and the procedure used in this study were fully described in previous publications from our laboratory [14, 15]. Briefly, the number of intratumor vessels throughout the tumor volume was defined as the mean number of vessels evaluated in both longitudinal and transversal planes. Then evaluation of tumor microvasculature was performed with a specific probe (emission at 2.6 MHz, reception at 5.2 MHz) (PLT-604AT, Toshiba, France), based on contrast microbubble detection (VRI, Vascular Recognition Imaging, Toshiba, France) after a 100 *μ*L bolus injection of Sonovue (Bracco, Italy). The contrast uptake was recorded during 3 minutes and quantified over the whole tumor section using dedicated software (CHI-Q®, Toshiba, France). Semiquantitative perfusion parameters were extracted from the time-intensity curves as described previously [15]: peak intensity (PI) and whole area under the curve (AUC).

**Figure 2:**
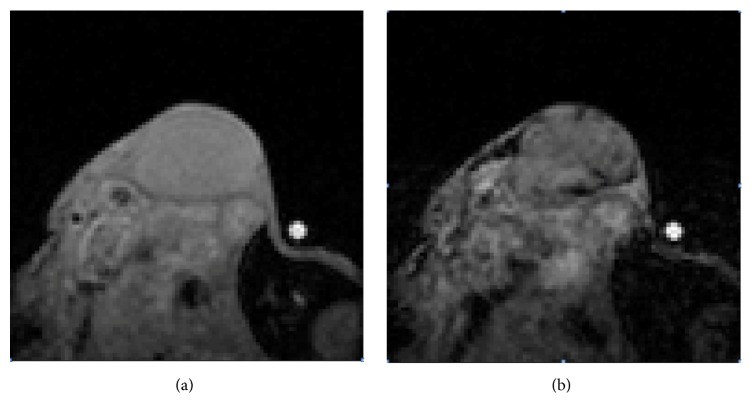
Measure of Dynamic Susceptibility Contrast- (DSC-) MR during 1 h through the repetition of *T*2^*∗*^ acquisitions. (a) Preinjection and (b) 1 hour after injection.

MR imaging was then performed using a clinical 1.5 T (Philips Achieva, CIERM) equipped with a 60 cm bore and a conventional 23 mm diameter surface coil in reception. The MRI contrast agent was the P04000 (Chematech/Guerbet, France), a contrast agent targeting *ανβ*3 integrin overexpressed on the neovessels. P04000 is a nanoemulsion (referred to as E1 in patent application US 2015/0320889 A1 untitled “vectorised magnetic emulsion” filed on Nov. 12, 2015), functionalized with RGD binding *ανβ*3, containing iron oxide particles designed for *T*2^*∗*^w MR sequences (susceptibility weighted imaging). Relaxation rate *R*2^*∗*^ measurements were done through a 3D *T*2^*∗*^-weighted gradient echo multiecho sequence, selected owing to its sensitivity to USPIO- (ultrasmall superparamagnetic iron oxide contrast agent-) induced apparent relaxation effects. Dynamic Susceptibility Contrast- (DSC-) MR (Figure 2) was performed during one hour from the contrast agent injection, through the repetition of *T*2^*∗*^ mapping (3D 0.5 mm isotropic sequence TR/TE/dTE = 90/5.9/9.7 msec, 6 echoes, 0.3 × 0.3 × 0.5 mm^3^ reconstruction voxel size, 220 Hz/pix, and Tacq = 4.2 min), before and after intravenous injection of 100 *μ*mol Fe/kg USPIO-based nanoemulsion per mouse. All images were processed using Matlab software (2011B, Mathworks, Natick, MA, USA). To reconstruct relaxation rate *R*2^*∗*^ (=1/*T*2^*∗*^) maps, the 6 echo signals *S*(TE) were fitted voxel-wise using a nonlinear least-squares algorithm to a 2-parameter exponential decay, based on the following equation: *S*(TE) = *A* · *e*^−TE·*R*2^*∗*^^, where *A* and *R*2^*∗*^ were the unknown parameters. The mean pre- and postvariations Δ*R*2^*∗*^ (*R*2^*∗*^pre − *R*2^*∗*^post) were calculated and followed for 1 hour to quantify binding to the targeted receptor. Indeed, in a simplistic model, *R*2^*∗*^ is modified linearly with contrast agent quantity such that Δ*R*2^*∗*^ is directly proportional to the binding.

### 2.4. Immunohistochemistry

**Figure 3:**
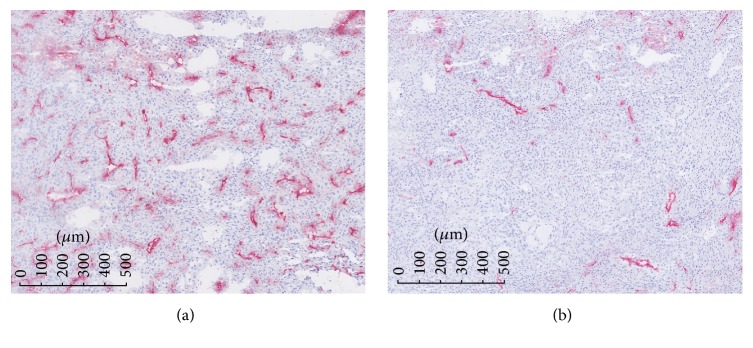
Images of CD31-stained tumor sections (magnification ×20) from (a) control group and (b) bevacizumab group.

Immunohistochemical (IHC) analysis was performed on the tumors extracted from both control and treated groups at D4 after the second imaging session. First, tumor xenograft tissues were embedded in an OCT gel like medium (Cryomatrix OCT compound, Thermo Fisher Scientific, USA), consisting of polyethylene glycol and polyvinyl alcohol, and then frozen in liquid nitrogen. Then, all the samples were cut into 7-8 *μ*m thick sections as close as possible to the maximal transverse section imaged by DCE-US and MRI. Hematoxylin, eosin, and safran (HES) stained sections were realized to verify tissue integrity. About 2 slides from each tumor were incubated with a monoclonal rat anti-mouse CD31 antibody (rat anti-mouse CD31 1 : 200, clone mec13-3, BD Pharmingen, USA) for immunohistochemical detection of endothelial cells (Figure 3). The whole tumor tissue sections from each animal were digitized using a slide scanner Nanozoomer (Hamamatsu). Quantification of CD31-stained microvessels (mean value) was achieved on the whole sections using image analysis software (Calopix, Tribvn, France).

### 2.5. Statistical Analysis

Nonparametric tests were performed in this study due to the number of samples per group. Wilcoxon or Kruskal-Wallis tests were used to compare data from the different groups of mice. Data from the same group were compared using Wilcoxon test for paired samples.

## 3. Results

**Figure 4:**
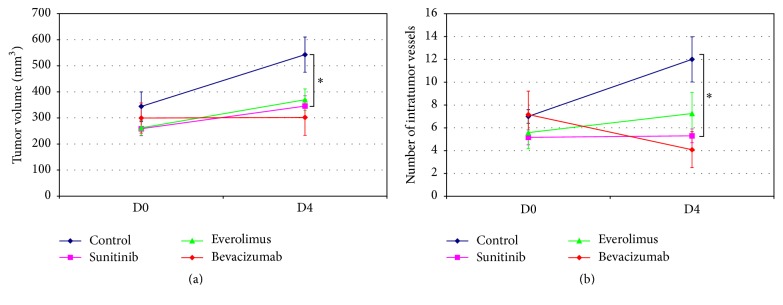
(a) Tumor volume evaluated by ultrasonography in mm^3^ at baseline (day 0, D0) and 4 days after treatment initiation (day 4, D4) measurements. (b) Number of intratumor vessels measured by power Doppler imaging for D0 and D4 measurements. Mean values ± standard errors. ^*∗*^*p* < 0.01.

Six mice per group were evaluated by both DCE-US and molecular MRI at D0 and D4. One mouse from the sunitinib group died during anesthesia procedure before the second imaging evaluation at D4.  Figure 4 presents the tumor growth (a) and the number of intratumor vessels (b) evolution between D0 and D4 for the 4 groups measured by ultrasonography. No significant difference in tumor volumes was observed between groups at D0 whereas at D4 control group exhibited higher tumor volumes compared to treated groups (*p* = 0.01). Tumor volume is stable for bevacizumab group between D0 and D4 and a slowed growth was observed for everolimus and sunitinib groups, compared to the control. The number of vessels estimated by power Doppler imaging was not significantly different for the 4 groups at D0, whereas lower values were observed for treated groups at D4 compared to the control group (*p* = 0.008). Bevacizumab group exhibits lower number of vessels at D4 compared to D0 but this decrease was not significant.

**Figure 5:**
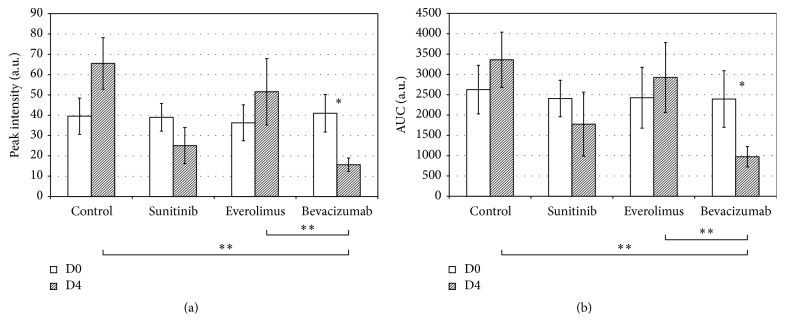
Mean values ± standard errors (a.u., arbitrary units) for peak intensity (PI) and area under the curve (AUC) measurements for the four groups at both baseline (day 0, D0) and 4 days after treatment initiation (day 4, D4). ^*∗*^*p* < 0.05, ^*∗∗*^*p* < 0.01.

Perfusion parameters, PI and AUC, are shown in Figure 5. These two parameters have been quantified because they reflect the modifications of enhancement during therapy and have been proven to be a validated criterion to predict therapy efficacy in preclinical and in clinical studies [14, 16]. No significant difference for PI (*p* = 0.9) and AUC (*p* = 0.9) was observed at D0 indicating homogeneous groups in terms of microvasculature and homogeneous bolus contrast injections between mice.

Four days after the start of treatment, both perfusion parameters exhibited higher mean values compared to values at D0, for control and everolimus groups, but these variations were not significant. On the contrary, a decrease was observed at D4 for groups who received sunitinib and bevacizumab administration, but the difference was only significant for bevacizumab group for both PI (*p* = 0.03) and AUC (*p* = 0.04). At D4, mean values for bevacizumab group were also significantly different from both everolimus group for PI (*p* = 0.006) and AUC (*p* = 0.008) and control group for PI (*p* = 0.004) and AUC (*p* = 0.008). These findings are in agreement with a clinical study [17] where dynamic changes in tumor vascularity were observed as early as 3 days after bevacizumab administration in patients with advanced hepatocellular carcinoma (HCC) and may be predictive of tumor response at 2 months, progression-free survival, and overall survival.

**Figure 6:**
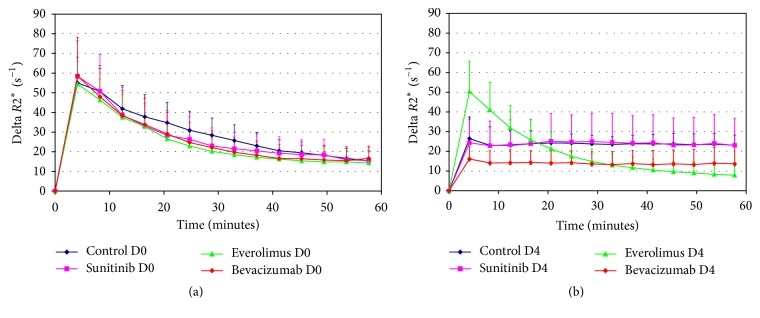
Delta *R*2^*∗*^ (s^−1^) profiles (mean values ± standard deviations) as a function of time during the 1 h measurements for the four groups at baseline (day 0, D0) (a) and 4 days after treatment initiation (day 4, D4) (b).

In addition to DCE-US measurements, therapy efficacy was evaluated by molecular MR imaging by measuring Δ*R*2^*∗*^ 1 h after the injection of the specific contrast agent P04000 (Guerbet, France) and thus the binding to *ανβ*3 receptor.  Figure 6 presents the profiles of mean values of Δ*R*2^*∗*^ averaged over all the mice (*n* = 6 per group) as a function of time for all the groups at both D0 (a) and D4 (b). Mean values ± standard deviations of Δ*R*2^*∗*^ (s^−1^) measured at 1 h are reported in Table 1. As for DCE-US measurements, no significant difference at D0 was found between the four groups (*p* = 0.99) or within mice from the same group indicating a good homogeneity between measurements and contrast agent injections.

At D4, Δ*R*2^*∗*^ was significantly different between groups (*p* = 0.04). Everolimus group exhibited lower values at D4 compared to D0 and significant differences were found for everolimus (*p* = 0.009) and bevacizumab (*p* = 0.048) groups at D4 compared to control group.

**Figure 7:**
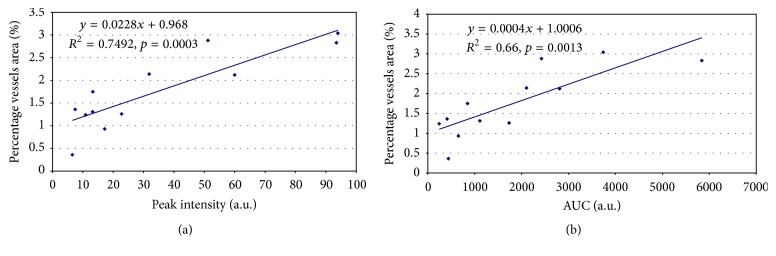
Correlation of percentage vessels area (%) quantified using CD31-staining on tumor tissue section versus dynamic contrast enhanced ultrasonography parameters, peak intensity, PI (a.u., arbitrary units) (a), and area under the curve, AUC (a.u., arbitrary units) (b). The linear regression equation with the coefficient of determination *R*^2^ and the *p* value are indicated.

Quantification of CD31-stained microvessels was performed using image analysis software (Calopix, Tribvn, France). Microvascular density was determined as a percentage of vessel area compared to total tissue area. No significant difference was found for the percentage of vessel tissue area (mean values ± standard deviations) in everolimus group (2.14 ± 0.40%) compared to control group (2.72 ± 0.41%), but this parameter was significantly reduced (*p* < 0.01) in both sunitinib (1.28 ± 0.05%) and bevacizumab groups (1.13 ± 0.52%). Finally the percentage of vessels quantified by IHC was compared to DCE-US and MRI data. No significant correlation was observed with MRI. The correlations between the percentages of vessels area with DCE-US parameters are illustrated in Figure 7 for both PI (a) and AUC (b). Significant correlations were observed for both PI (*R* = 0.87, *p* = 0.0003) and AUC (*R* = 0.81, *p* = 0.0013).

## 4. Discussion

In this study, we demonstrated, in an in vivo animal experiment setting, *α*v*β*3-targeted MRI ability to detect the early microvasculature response to everolimus and bevacizumab while DCE-US only detected response to bevacizumab.

Neoangiogenesis is a paramount hallmark for tumor development and it is the direct or indirect target of systemic therapies used in metastatic RCC. The application of DCE-US to predict response to antiangiogenic treatment for metastatic RCC had already been explored in several clinical trials that emphasized its potential to differentiate responders versus nonresponders at an earlier stage than conventional methods [18]. DCE-MRI evaluation has also been reported in studies where *K*_trans_ was the most frequently used parameter [19–21]. It was reported in a randomized trial as a pharmacodynamic biomarker for sorafenib response [19]. However, those trials only assessed response to antiangiogenic therapies, per se bevacizumab or tyrosine kinase inhibitors (TKI): sunitinib and sorafenib.

Unlike bevacizumab or TKI, mammalian targets of rapamycin inhibitors are not directly targeting the VEGF pathway but indirectly interact with angiogenesis by controlling Hypoxia Inducible Factors (HIF) mRNA translation [22]. They are indicated for poor prognosis mRCC (temsirolimus) or after failure of TKI (everolimus) [23]. Early assessment of their efficacy would be particularly valuable in clinical practices to avoid potentially inefficient treatment to the patients.

The endothelial integrin *α*v*β*3 interacts with VEGFR2 to drive neoangiogenesis after upstream activation by tumor cells [24]; it also plays a role in anchorage-independence acquisition by tumor cells, a key feature for tumor progression [25]. In 1998, Sipkins et al. first reported in vivo tumor angiogenesis monitoring using *α*v*β*3-targeted MRI [26]. To our knowledge, molecular MRI in general and this technique more particularly have never been applied to evaluate response to targeted therapy in RCC. Here, we report that the difference in rate of signal decay per second (Δ*R*2^*∗*^ s^−1^) before and after injection of *α*v*β*3-targeted contrast agent was significantly lower among bevacizumab and everolimus treatment groups versus placebo group. This finding was measured as early as 4 days after treatment initiation and was correlated with the tumor growth rate. This result suggests an early vessel maturation modification after administration of these agents. Signal attenuation after targeted contrast agent could be a potential biomarker candidate, in association with *K*_trans_ for an early assessment of tumor's response to the newly introduced targeted agent. MRI presents several advantages as an imaging technique: no ionizing radiation, DCE sequences can be combined with an evaluation of water molecules mobility (diffusion), and MR spectroscopy allows an evaluation of the chemical composition of the tissues and detect hypoxic regions [27]. These multiparametric settings deliver multiple information on the tumor from a single procedure. However, MRI remain more time-consuming than US and CT implying a more restricted access to this imaging platform, standardization of the procedures is more challenging, and the correlation between imaging signal and marker concentration is not as straightforward as it is with CT measurements [28].

One key point of this study is the 4 days of early detection in imaging signal. In clinical practices, evaluation of anticancer treatment response broadly relies on the tumor-size based response evaluation criteria in solid tumors (RECIST) [29]. This consensual criterion is based on the measurement of the greatest diameter of the tumor (or metastases). Response can be complete (absence of disease), partial (more than 30% decrease), progressive disease (more than 20 times increase), or stable disease (in between). The problem is the delay between treatment induction and its effect on tumor morphology and mass shrinkage. During this delay it is impossible for the clinician relying on RECIST to differentiate responders from nonresponders. Moreover, necrosis or fibrosis often induced by targeted therapies might not lead to size decrease while it should not be considered as active vital tumor. Metabolic changes induced by targeted therapy in the tumor and more specifically neovessels development and induction signals precede size reduction. This earlier detection of treatment efficacy might allow a better treatment tailoring for the patient who would benefit from instantaneous switch in his therapeutic strategy. Another benefit awaited with this new technique is the potential financial cost saved by preventing useless expensive targeted drugs prescriptions. Although not performed here and beyond the scope of this study, following the evolution of the *α*v*β*3 signal attenuation in parallel with tumor growth would allow evaluating the ability of this marker to detect treatment resistance.

This study presents some limitations. As a preclinical trial, the number of mice included in analyses is restricted. We used A498 cell-line based xenograft model with subcutaneous implantation. Although this model is commonly used, discrepancies with clinical trials had often been reported. Final conclusions are still to be confirmed by clinical validation.

## 5. Conclusion

We report an early detection of neoangiogenesis modification after induction of targeted therapies, using DCE-US or *α*v*β*3-targeted MRI. We consider these outcomes should encourage clinical trial developments to further evaluate the potential of this molecular MRI technique.

## Figures and Tables

**Table 1:** Mean values ± standard deviations of Δ*R*2^*∗*^ (s^−1^) evaluated at 1 h for the four groups at both baseline (day 0, D0) and 4 days after treatment initiation (day 4, D4).

	Δ*R*2^*∗*^ (s^−1^)	
	D0	D4
Control	15.3 ± 5.2	23.1 ± 5.1
Sunitinib	15.4 ± 6.7	23.0 ± 13.8
Everolimus	14.3 ± 5.6	7.9 ± 4.8
Bevacizumab	16.6 ± 6.3	13.6 ± 8.9
